# The Role of Herbal Medicine as Anti-Cancer Medicine: From the Claim to Truth

**DOI:** 10.22086/gmj.v0i0.1179

**Published:** 2018-04-08

**Authors:** Hoda Aryan

**Affiliations:** ^1^Young Researchers and Elite Club, Tehran Medical Sciences Branch, Islamic Azad University, Tehran, Iran; ^2^Medical Students’ Scientific Association (MSSA), Tehran Medical Sciences Branch, Islamic Azad University, Tehran, Iran

**Keywords:** Cancer, Herbal Medicine, Complementary, Anti-cancer

## Dear Editor,

One of the main causes of death in the world is cancer, so that has involved more than one-third of the population, and is considered as the cause of more than 20 percent of all deaths in the world [1]. More than 100 different types of cancer have been identified. Regarding the International Agency for Research on Cancer (IARC) reports, the most commonly diagnosed cancers worldwide were those of the lung (1.8 million, 13.0% of the total), breast (1.7 million, 11.9%), and colorectum (1.4 million, 9.7%) [2].

If the cancer is detected in its early stages, surgery or radiation therapy can be used to treat, but advanced cancer treatment needs chemotherapy. Although chemotherapy drugs are effective, their use is associated with abundant adverse effects and drug resistance [3].

Herbal remedies have been used over the centuries to treat a variety of diseases. Most of these treatments as alternatives or supplements to consider a way that helps patients to have better physical and mental status. Currently, many in-vitro and in vivo studies indicated the beneficial effect of medicinal plants and their bioactive compounds could induce the apoptosis in neoplastic cells [4-8].

Previous studies showed the anti-cancer activity of the ten widely used herbs that are commonly used in the context of cancer by patients in the Middle East: *Olea Europea* (Olive), *Nigella Sativa* (Black Seeds), *Crocus Sativus* (Saffron), *Punica Granatum* (Pomegranate), *Urtica Dioica* (Nettle), *Allium Sativum L*. (Garlic), *Allium Cepa* (Onion), *Curcuma longa* (Curcumin), *Arum Palaestinum* (Palestinian Arum), and *Vitis Vinifera* (Grapes) [9, 10].

However, researchers have believed to confirm the usefulness of herbal medicines in the treatment or prevention of the cancer is essential to conduct extensive clinical trials to determine what plants can be used alongside conventional therapeutic methods for cancer treatment.

Nowadays, more than 25% of drugs used during the last 20 years are directly derived from plants, while the other 25% are chemically altered natural products. Still, only 5–15% of the approximately 260,000 higher plants have ever been investigated for bioactive compounds [3, 4]. The advantage of using such compounds for cancer treatment is their relatively low/non-toxic nature.

According to the WHO, 80 percent of the world benefit from traditional treatment. Sixteen percent of drugs approved by the FDA in the years 1984 to 1994 have been obtained from natural resources, especially plants [4]. Vinca Alkaloids (Vinblastine and Vincristine), Taxanes, podophyllotoxin are the best known FDA approved plant-derived anticancer agents.

Vinblastine and Vincristine are the first herbal anti-cancer medicine that introduced in 1960 which are widely used to treat breast cancer, Hodgkin’s Lymphoma, William’s tumor, lymphoblastic leukemia and Kaposi’s sarcoma [4-6].

In 1992, Taxanes, the most efficient anti-tumor agent, was approved by FDA to treat breast, head, and neck, prostate, and gastric cancers [4, 6, 7].

Etoposide and teniposide are two active and semi-synthetic compounds of Epipodophyllotoxin that is approved by FDA for the treatment of choriocarcinoma, lung cancer, ovarian and testicular cancers, lymphoma and acute myeloid leukemia. [8] Considering the fact that little is known about efficacy and safety of herbal products, and not paying attention to commonly used products setting, further research can improve appropriate use of plant products drastically.
